# Bimatoprost 0.01% in treatment-naïve patients with open-angle glaucoma or ocular hypertension: an observational study in the Korean clinical setting

**DOI:** 10.1186/1471-2415-14-160

**Published:** 2014-12-17

**Authors:** Ki Ho Park, Susan Simonyi, Chan Yun Kim, Yong Ho Sohn, Michael Scott Kook

**Affiliations:** Department of Ophthalmology, Seoul National University Hospital, 101 Daehak-ro, Jongno-gu, Seoul, 110-744 Korea; Allergan Singapore Pte, Ltd, Singapore, Singapore; Department of Ophthalmology, Yonsei University, College of Medicine, Seoul, Korea; Kim’s Eye Hospital, Seoul, Korea; Eye Center, Department of Ophthalmology, Asan Medical Centre, University of Ulsan, College of Medicine, Seoul, Korea

**Keywords:** Bimatoprost, Glaucoma, Hyperemia, Intraocular pressure, Prostaglandin analog, Prostamide

## Abstract

**Background:**

This study evaluates the efficacy and tolerability (ie, occurrence and severity of hyperemia) of bimatoprost 0.01% in treatment-naïve patients with open-angle glaucoma (OAG) or ocular hypertension in the Korean clinical setting.

**Methods:**

In this multicenter, open-label, observational study, treatment-naïve patients with OAG, including patients with normal-tension glaucoma (NTG, defined as IOP ≤21 mm Hg), or ocular hypertension received bimatoprost 0.01% once daily. Hyperemia was assessed at baseline and weeks 6 and 12, graded by a masked evaluator using a photonumeric scale (0, +0.5, +1, +2, +3), and grouped as (0 to +1) and (+2 to +3). Shifts between groupings were reported as no change, improved ([+2 to +3] to [0 to +1]), or worsened ([0 to +1] to [+2 to +3]). Other adverse events were monitored. Mean IOP changes from baseline at weeks 6 and 12 were reported. Supplemental analyses were conducted for IOPs >21 versus ≤21 mm Hg.

**Results:**

Of 295 treatment-naïve patients included in the intent-to-treat/safety population, 73 (24.7%) had baseline IOP >21 mm Hg (mean, 25.7 ± 5.0 mm Hg) and 222 (75.3%) had baseline IOP ≤21 mm Hg (mean, 16.3 ± 3.0 mm Hg); 96.3% had hyperemia graded none (36.3%) to mild (17.3%). At week 12, hyperemia was graded none to mild in 83.7% (n = 220). Worsening occurred in 12.3% of patients by week 6 and 12.7% by week 12. Small improvements occurred in 0.8% and 0.5% of patients at weeks 6 and 12, respectively. Hyperemia scores were generally low and the majority of patients had no change in severity during the study. Mean IOP at weeks 6 and 12 was reduced to 16.4 ± 4.0 mm Hg (-34.5%; *P* < 0.0001) and 16.7 ± 3.9 mm Hg (-32.0%; *P* < 0.001) in the baseline-IOP >21 mm Hg group versus 13.3 ± 2.6 mm Hg (-17.8%; *P* < 0.001) and 13.7 ± 2.8 mm Hg (-15.9%; *P* < 0.001) in the baseline-IOP ≤21 mm Hg group, respectively.

**Conclusions:**

In treatment-naïve patients, bimatoprost 0.01% induced low shifts in worsening of hyperemia and significant reductions in IOP, regardless of baseline IOP.

**Trial registration:**

Clinical trial registration number: NCT01594970

## Background

Glaucoma is the second leading cause of blindness globally [1], and estimates of prevalence suggest that glaucoma-induced bilateral blindness will affect 11.1 million people in 2020 [2]. Research has shown that the prevalence and characteristics of glaucoma vary by geography and race [3]. For example, prevalence of primary open-angle glaucoma (POAG) is reportedly higher in Africa, Japan, and Latin America than China or India [2]. In a population-based survey study of residents at least 50 years of age in Oeso-myeon, Sangju City, South Korea, the prevalence of glaucoma was 3.4% (95% confidence interval, 2.1–4.8) [4], similar to that seen in Japan [5]. Furthermore, the overwhelming majority of cases (94%) were open-angle glaucoma (OAG) with low/normal intraocular pressure (IOP ≤21 mm Hg), ie, normal-tension glaucoma (NTG) [4], which appears to be a common feature of glaucoma in Asian patients relative to those in Western countries [5–7].

Elevated IOP is a key risk factor for the progression of glaucoma, and lowering IOP is the focus of glaucoma management strategies, including NTG. Based on data from randomized, controlled clinical trials demonstrating IOP-lowering efficacy, prostaglandin analogs are considered first-line treatment options for patients with glaucoma or ocular hypertension (OHT). Bimatoprost 0.03%, a prostamide, is an effective IOP-reducing treatment that is well tolerated with long-term use [8–10]; it also has demonstrated superior IOP-lowering effects compared with other prostaglandin analogs used as monotherapy [8, 9, 11, 12].

Prostaglandin analogs and prostamides are generally well tolerated, but some patients experience ocular adverse events, the most common being hyperemia. Although generally transient, these adverse events may affect patient adherence and contribute to treatment discontinuation. In response to these concerns, a 0.01% formulation of bimatoprost was developed to improve tolerability. In a randomized, controlled study, bimatoprost 0.01% was shown to be equivalent to bimatoprost 0.03% in lowering IOP over a 12-month treatment period, while also demonstrating improved tolerability, particularly in terms of less frequent and less severe conjunctival hyperemia [13]. In the recent 12-week, open-label, Canadian Lumigan RC Early Analysis Review (CLEAR) study, which was designed to replicate the real-world clinical setting, bimatoprost 0.01% significantly reduced IOP from baseline in treatment-naïve patients [14]. Only 6% of these patients developed moderate to severe hyperemia during therapy, and most experienced no or very mild hyperemia [14]. Similar results were seen in a prospective, observational study in Germany, with significant IOP reductions in both treatment-naïve and previously treated patients, and hyperemia reported in only 1.2% of patients [15].

Given the potential geographic and racial differences in the presentation of glaucoma, the observational Asia Pacific Patterns from Early Access of Lumigan 0.01% (APPEAL) study was initiated to assess the external validity of the controlled trials of bimatoprost 0.01% in an Asian population. “APPEAL Korea” evaluated the tolerability and efficacy of bimatoprost 0.01% in the clinical practice setting in Korea. We report herein the results of this observational study in the subgroup of treatment-naïve patients with OAG or OHT who were enrolled in APPEAL Korea, with supplemental efficacy analyses based on baseline IOP. Because this study was designed without restrictions imposed by rigorous eligibility criteria, it closely parallels the real-world clinical setting in Korea.

## Methods

### Study design and patients

This open-label, 12-week, evaluator-masked, noncomparative, observational clinical evaluation of bimatoprost 0.01% in patients with OAG, including NTG, or OHT (ClinicalTrials.gov identifier: NCT01594970; registered on May 8, 2012) was conducted in 29 clinical centers in Korea between April 2012 and January 2013. All study participants assigned to treatment were Korean. All investigators obtained appropriate institutional review board approvals from their respective institutions (see Acknowledgements) before commencing the study. Study participants provided written informed consent prior to any study-related procedure or change in treatment. The study was conducted in accordance with the principles set forth in the Guidelines for Good Clinical Practice and the Declaration of Helsinki (and its amendments) [16].

Patients included were at least 20 years of age and had been diagnosed before screening with OHT or OAG (including NTG) by the treating physician according to standard of care in their practice. OAG was defined as an eye with glaucomatous optic nerve head change and corresponding glaucomatous visual field defects, and requirement for treatment with bimatoprost 0.01%. Patients were excluded if they had hypersensitivity to any prostaglandin analog or any component of the study medication; presence of any other abnormal ocular condition or symptom preventing study participation. Women who were pregnant, planning a pregnancy, nursing, or of child-bearing potential and were not using a reliable form of contraception were also excluded.

This analysis focuses on patients who were treatment naïve prior to the study and received bimatoprost 0.01% monotherapy as study treatment. A similar analysis of patients who switched from a prior IOP-lowering medication to bimatoprost 0.01% (administered as monotherapy or adjunctive therapy) will be presented elsewhere.

### Treatment

Bimatoprost 0.01% was obtained by the patient or study center through commercial means and not as investigational study drug provided by Allergan, Inc. At the baseline visit, patients were instructed to self-instill their medication into the affected eye(s) each evening. In patients in whom both eyes were eligible for inclusion, both were treated, but only the one with the highest baseline IOP was included in the analysis. If the baseline IOP was the same in both eyes, the right eye was included as the study eye.

### Outcomes

Outcome variables were assessed at baseline and weeks 6 and 12 (at 10 am ± 2 hours). The primary outcome variable was the week-12 incidence and severity of ocular hyperemia, assessed by a qualified evaluator masked to the patient’s previous treatment history. Hyperemia was graded using a 5-point photographic bulbar conjunctival hyperemia grading scale: 0, none, normal; +0.5 trace, trace flush reddish pink; +1, mild, mild flush reddish color; +2, moderate, bright red color; +3, severe, deep, bright, diffuse redness. Hyperemia grading was collapsed into 2 categories, ie, none to mild (ratings of 0, +0.5, or +1) and moderate to severe (ratings of +2 or +3), as described by other groups [14, 17]. The change in hyperemia grading from baseline at weeks 6 and 12 (for the collapsed grading categories) was summarized as improved, no change, or worsened.

Secondary efficacy endpoints were change in IOP from baseline and the percentage change in IOP from baseline at 6 and 12 weeks, using the same type of Goldmann applanation tonometer as was used for the baseline assessment. The examiners were instructed to perform IOP measurements in the morning at approximately the same time of the day (ie, 10 AM ± 2 hours) for a given patient throughout the study. Safety was assessed throughout the study in terms of reported ocular adverse events and their severity, seriousness, and relationship to the study medication. In addition, biomicroscopy was performed, visual acuity assessed, and the number of discontinuations due to adverse events was recorded. Every effort was made to contact each patient if they did not return for a scheduled visit and to document the patient outcome.

### Statistical analysis

All patients who received at least 1 dose of study medication were included in the efficacy analyses (intent-to-treat [ITT] population). Safety analysis was performed using the safety population, which was identical to the ITT population. Individual hyperemia scores reported for each of the 5 grades (ie, 0, +0.5, +1, +2, +3) were summarized as frequency counts and percentages for all visits, and change from baseline was reported at weeks 6 and 12. For statistical analysis, change in hyperemia grading was defined as a shift from one hyperemia grouping to another between baseline and week 6 or week 12 [14]. Patients with measures at baseline and each time point were included in this analysis. Treatment effect was assessed using a 2-sided McNemar test.

Change in IOP from baseline and percentage change in IOP from baseline were summarized at each visit using descriptive statistics. Changes from baseline in IOP and percentage changes from baseline at week 6 and week 12 were assessed using the 2-sided Student paired *t-*test.

Discontinuations due to adverse events were summarized using frequency and rates. The study was exploratory in nature and, as such, no formal sample size calculations were carried out.

## Results

### Patients

A total of 801 patients from 29 Korean centers were enrolled in APPEAL Korea. One enrolled subject failed to meet the inclusion criteria and was excluded from the study. Consequently, the overall ITT population comprised 800 patients: 543 (67.9%) received monotherapy with bimatoprost 0.01%, and 257 (32.1%) patients received bimatoprost 0.01% as adjunctive therapy with at least 1 other agent. Within the monotherapy group, there were 295 (54.3%) patients who were treatment naïve. Data from patients who switched to bimatoprost 0.01% monotherapy and those who received bimatoprost 0.01% as adjunctive therapy will be presented elsewhere.

Of the 295 treatment-naïve patients (ITT population), 220 completed the study. Of the 75 patients who discontinued, 54 (72.0%) were lost to follow-up, while 16 (21.3%), 1 (1.3%), and 4 (5.3%) discontinued due to ocular adverse events, other adverse events, or other reasons, respectively. Two patients were recorded as discontinuations despite completing the study as scheduled: 1 due to an ocular adverse event and 1 due to other reasons (non–adverse event-related), both occurring at the end of the study.

Patients in the treatment-naïve group had a mean ± standard deviation (SD) age of 58.0 ± 13.8 years, and 55.9% were male. Male patients were younger than female patients (mean, 55.6 years vs 61.0 years). Most patients were diagnosed with OAG (94.6%), and the remaining patients had OHT; 222 (75.3%) patients had NTG with baseline IOP ≤21 mm Hg (mean IOP = 16.3 ± 3.0 mm Hg). The majority of patients had been newly diagnosed with OAG or OHT (42.0%), or diagnosed for less than a year (41.7%). The right eye was designated the study eye in 206 (69.8%) patients.

Diabetes (14.6%) and hypertension (30.2%) were the most common comorbid conditions among the 141 (47.8%) patients with at least 1 comorbid medical condition. One patient reported a history of asthma, a comorbidity that may have affected the occurrence of hyperemia. A total of 85 (28.8%) patients received at least 1 concomitant medication during the study. Anti-inflammatories or antihistamines used by patients during the study were systemic cyclosporine, olopatadine, and zaltoprofen, as well as ophthalmic fluorometholone, ketorolac, neomycin/dexamethasone, and neomycin/polymixin/dexamethasone (n = 1 each). Less than 1% of patients had corneal staining/erosion on biomicroscopy.

### Hyperemia

**Figure 1:**
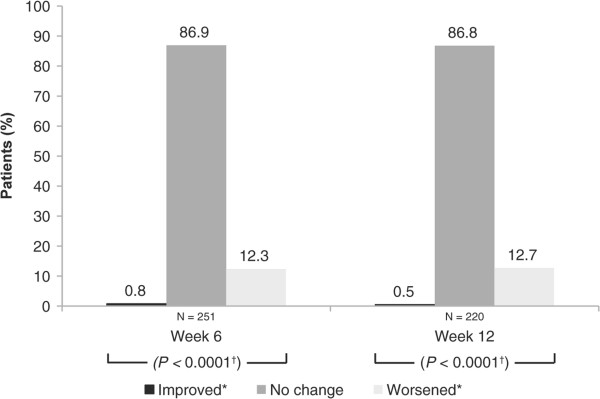
**Shift from baseline in hyperemia grading for all treatment-naïve patients.** *Improved: +2, +3 to 0, +0.5, +1; worsened: 0, +0.5, +1 to +2, +3. ^†^McNemar analysis.

At week 12, 16.4% of patients experienced moderate or severe hyperemia (Table 1). When hyperemia severity was grouped according to the 2 severity categories, 83.6% of patients had no or mild hyperemia (Table 2). Notably, the majority of patients who had none to mild hyperemia at baseline experienced no change or improved at both weeks 6 and 12 (Table 3). Moreover, most patients who had moderate to severe hyperemia at baseline improved at weeks 6 and 12 (Table 3). After collapsing the data to 2 severity categories (as reported by other groups) [14, 17], most patients showed no shift in hyperemia grading at week 6 (86.9%) and week 12 (86.8%) (Figure 1). Hyperemia worsened in 12.3% of patients by week 6 and in 12.7% of patients by week 12, but small percentages of patients showed improvements in hyperemia grading at week 6 (0.8%) and week 12 (0.5%). The changes in the collapsed severity scores from baseline to weeks 6 and 12 were significant (*P* < 0.0001) (Figure 1).

**Table 1:** **Occurrence and severity of ocular hyperemia by severi** **ty grade**

	Baseline	Week 6	Week 12
No. (missing^a^)	295 (0)	251 (44)	220 (75)
Hyperemia grading, n (%)			
0 (none, normal)	107 (36.3)	39 (15.5)	40 (18.2)
+0.5 (trace)	126 (42.7)	76 (30.3)	71 (32.3)
+1 (mild)	51 (17.3)	97 (38.6)	73 (33.2)
+2 (moderate)	10 (3.4)	32 (12.7)	31 (14.1)
+3 (severe)	1 (0.3)	7 (2.8)	5 (2.3)

^a^Patients for whom hyperemia grading data were unavailable at scheduled visit.

**Table 2:** **Occurrence and severity of ocular hyperemia by two severity categories**

	Baseline	Week 6	Week 12
No. (missing^a^)	295 (0)	251 (44)	220 (75)
Hyperemia category, n (%)			
None to mild (0, +0.5, +1)	284 (96.3)	212 (84.5)	184 (83.6)
Moderate to severe (+2, +3)	11 (3.7)	39 (15.5)	36 (16.4)

^a^Patients for whom hyperemia grading data were unavailable at scheduled visit.

**Table 3:** **Shift from baseline in hyperemia severity grading at follow-up**

Baseline	Week 6, n (%)				
	None	Trace	Mild	Moderate	Severe
0 (none)	34 (13.5)	5 (2.0)	0 (0)	0 (0)	0 (0)
+0.5 (trace)	26 (10.4)	41 (16.3)	8 (3.2)	1 (0.4)	0 (0)
+1 (mild)	21 (8.4)	49 (19.5)	26 (10.4)	1 (0.4)	0 (0)
+2 (moderate)	11 (4.4)	7 (2.8)	7 (2.8)	7 (2.8)	0 (0)
+3 (severe)	2 (0.8)	2 (0.8)	2 (0.8)	0 (0)	1 (0.4)
Missing	13	22	8	1	0
**Baseline**	**Week 12, n (%)**				
0 (none)	33 (15.0)	6 (2.7)	1 (0.5)	0 (0)	0 (0)
+0.5 (trace)	20 (9.1)	38 (17.3)	13 (5.9)	0 (0)	0 (0)
+1 (mild)	22 (10.0)	35 (15.9)	15 (6.8)	1 (0.5)	0 (0)
+2 (moderate)	6 (2.7)	11 (5.0)	7 (3.2)	7 (3.2)	0 (0)
+3 (severe)	0 (0)	3 (1.4)	1 (0.5)	0 (0)	1 (0.5)
Missing	26	33	14	2	0

### Intraocular pressure

**Figure 2:**
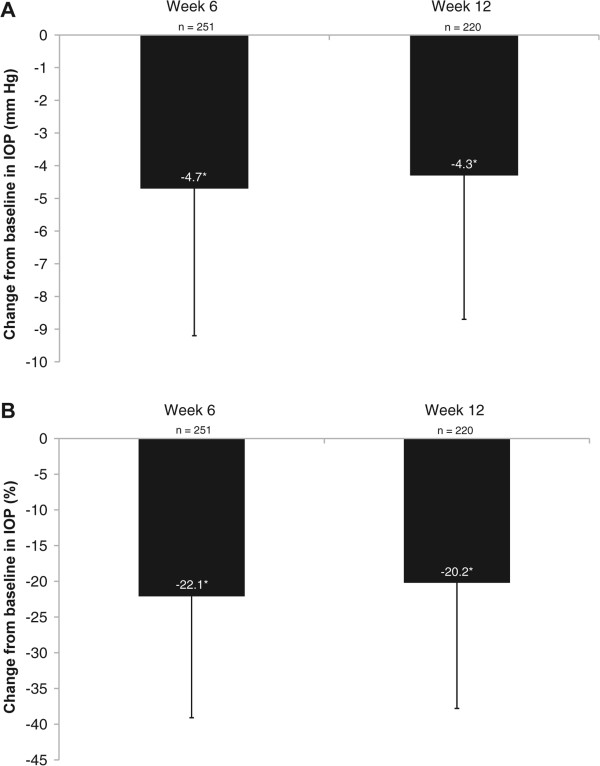
**Absolute (A) and percentage (B) IOP reductions from baseline for all treatment-naïve patients.** Data are presented as mean ± SD. *P < 0.0001 (1-sample *t*-test). IOP = intraocular pressure.

The overall mean baseline IOP ± SD was 18.6 ± 5.4 mm Hg. Following initiation of bimatoprost 0.01% therapy, IOP decreased to a mean of 14.1 ± 3.3 mm Hg at week 6 and 14.5 ± 3.4 mm Hg at week 12, which were statistically significant reductions from baseline (both *P* < 0.0001) (Figure 2A). These changes corresponded to statistically significant mean IOP reductions from baseline of 22.1% at week 6 and 20.2% at week 12 (both *P* < 0.0001) (Figure 2B).

**Figure 3:**
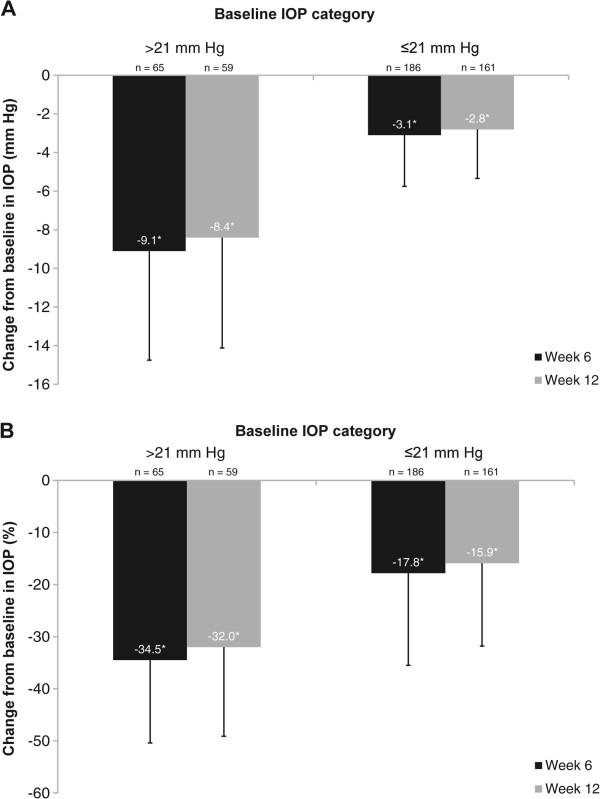
**Absolute (A) and percentage (B) IOP reductions from baseline for treatment-naïve patients, by baseline IOP.** Data are presented as mean ± SD. *P < 0.0001 (1-sample *t*-test). IOP = intraocular pressure.

Among the 73 patients (24.7%) with POAG/OHT and a baseline IOP >21 mm Hg, mean ± SD IOP at baseline was 25.7 ± 5.0 mm Hg, which decreased to 16.4 ± 4.0 mm Hg at week 6 (34.5% reduction) and 16.7 ± 3.9 mm Hg at week 12 (32.0% reduction) following bimatoprost treatment (both *P* < 0.0001 vs baseline) (Figure 3). Significant reductions from baseline were also observed in the patients with NTG. Mean (± SD) IOP was reduced to 13.3 ± 2.6 mm Hg at week 6 (17.8% reduction; *P* < 0.001) and 13.7 ± 2.8 mm Hg at week 12 (15.9% reduction; *P* < 0.001) (Figure 3).

### Safety

A total of 68 treatment-emergent adverse events were reported by 58 patients (19.7%). Fifty-seven ocular adverse events reported by 49 (16.6%) patients were considered treatment-related. No serious treatment-related adverse events were reported, but 1 serious adverse event unrelated to treatment was reported (malignant lung neoplasm). The most common treatment-related adverse events were hyperemia (7.1%) and skin hyperpigmentation (3.1%) (Table 4). Corneal staining/erosion were rated as no, trace, mild, moderate, or severe on biomicroscopy in 90.5%, 6.4%, 2.4%, 0.7%, and 0% of patients at baseline, and in 65.8%, 6.8%, 1.4%, 0.7%, and 0.3% at 12 weeks, respectively.

**Table 4:** **Treatment-related adverse events reported by ≥1% of treatment-naïve patients**

	Patients, n (%)
Patients with ≥1 treatment-related adverse event(s)	47 (15.9)^a^
Treatment-related adverse event	
Hyperemia	21 (7.1)
Dry eye	3 (1.0)
Eye pruritus	4 (1.4)
Deepening of superior sulcus	7 (2.4)
Eye pain	4 (1.4)
Skin hyperpigmentation	9 (3.1)
Other (<1%)^b^	9 (3.1)

^a^All were ocular in nature.

^b^Blurred vision, corneal erosion, eyelash growth, ocular discomfort (all 2 patients each); allergic conjunctivitis, skin hypopigmentation (1 patient each).

## Discussion

APPEAL Korea was designed to further assess the tolerability and efficacy of bimatoprost 0.01% in a real-world setting outside the limits of the narrow eligibility criteria of a randomized clinical trial, thereby better replicating clinical practice. The results of this analysis demonstrate that daily treatment with bimatoprost 0.01% for 12 weeks was associated with low rates of hyperemia while significantly reducing baseline IOP in patients with OAG or OHT who were treatment naïve.

Use of a standardized hyperemia grading scale and classification of patients according to a collapsed, 2-category severity grading scale showed that most patients experienced no to mild hyperemia at both week 6 (84.5%) and week 12 (83.6%). The shift analysis demonstrated that over the 12 week-study, the majority of patients (86.9% at week 6 vs 86.8% at week 12) experienced no change in hyperemia severity relative to baseline during treatment. Less than 13% of patients experienced a clinically significant increase in hyperemia severity grading at weeks 6 and 12. These observations are consistent with those described elsewhere [13–15]. The similar 12-week, open-label study of bimatoprost 0.01% in patients from Canada with POAG or OHT (CLEAR study) indeed demonstrated similar results, with most patients showing no change in hyperemia severity grading; however, the percentage of patients who had an increase in hyperemia severity from baseline was about half that seen here [14].

The statistically significant IOP reductions from baseline at both assessment visits during treatment with bimatoprost 0.01% were consistent with other studies. However, in the similarly designed CLEAR study, IOP reductions from baseline of approximately 30% were observed compared with the mean 20.2% reduction reported here. Patients in the CLEAR study had a higher mean baseline IOP than patients in this analysis (23.5 ± 5.8 mm Hg vs 18.6 ± 5.4 mm Hg). When changes in IOP were assessed only in patients with a baseline IOP ≥21 mm Hg (mean baseline IOP 25.7 ± 5.0 mm Hg), the IOP reduction at week 12 was 32%, consistent with results from the CLEAR study. Observed mean and percentage IOP reductions from baseline at weeks 6 and 12 were greatest in patients with the highest mean baseline IOP, but nonetheless, statistically significant IOP reductions were achieved irrespective of the patient’s baseline IOP.

An important finding in this analysis was the statistically significant reductions in IOP observed in patients with NTG [18]. In Asian countries where the prevalence of NTG is much higher than in Western countries, the mean IOP is also typically lower. Studies indicate that in Japan, 92% of patients with OAG have an IOP ≤21 mm Hg [5, 6], whereas in South Korea, 77% of OAG cases have an IOP ≤21 mm Hg [7].

IOP reduction is the only therapeutic approach that has been shown to slow the progression of visual field loss in patients with NTG [19, 20]. Our study showed IOP reductions from baseline at 12 weeks of 15.9% in patients with IOP ≤21 mm Hg. A Japanese study in patients with NTG and an IOP ≤18 mm Hg showed a statistically significant mean IOP reduction of 19.9% after 12 weeks’ treatment with bimatoprost 0.03% [21]. Statistically significant reductions in IOP were also seen in patients with IOP <13 mm Hg [21]. Data from studies investigating other IOP-lowering medications in patients with NTG have demonstrated similar reductions in patients with low baseline IOP [22–25]. Our data are thus consistent with these previous findings and, to the best of our knowledge, are the first to describe the effects of bimatoprost 0.01% treatment in a group of patients in whom the baseline IOP in the majority (75%) was ≤21 mm Hg.

Bimatoprost 0.01% treatment was well tolerated in this population. Consistent with the safety profile described elsewhere [13–15], the most common treatment-related adverse events was ocular, specifically hyperemia. Only 1 non-ocular, non-treatment related, serious adverse event was reported.

This open-label study was designed to capture data in the real-world clinical setting in Korea, and the results support the external validity of findings from more rigorously controlled clinical trials. Nonetheless, open-label studies are not without limitations, and the lack of a control group cannot be ignored. In our study, 75 treatment-naïve patients (25.4%) discontinued study medication. The majority of these patients (72.0%) were lost to follow-up. As no reasons are given for the failure to follow-up in these patients, despite the attempt to contact them, the possibility exists that some of these patients were lost due to a lack of efficacy or the occurrence of adverse events. Although this could have affected the efficacy and safety findings reported here, only 17 (22.7%) patients discontinued due to an ocular adverse event, representing 5.8% of the overall treatment-naïve population. This latter discontinuation rate is consistent with the 4.4% of patients who discontinued due to an ocular adverse event in the CLEAR study [14].

## Conclusions

This open-label study, which replicates the use of bimatoprost 0.01% in the clinical setting in Korean patients with OAG or OHT, demonstrated low rates of hyperemia with most patients experiencing no increases in hyperemia severity during treatment. Reductions in IOP were seen irrespective of baseline IOP, although the largest decreases from baseline were seen in patients with highest baseline IOP. Significant IOP reductions were seen in patients with IOP associated with NTG, an important finding given the increased prevalence of NTG in Asian countries. Bimatoprost 0.01% would therefore be a suitable option for IOP lowering in these patients.

## Abbreviations

APPEAL: Asia Pacific Patterns from Early Access of Lumigan
CLEAR: Canadian Lumigan RC Early Analysis Review
IOP: Intraocular pressure
ITT: Intent-to-treat
NTG: Normal-tension glaucoma
OAG: Open-angle glaucoma
OHT: Ocular hypertension
POAG: Primary open-angle glaucoma
SD: Standard deviation.
